# Application of Mn_*x*_Fe_1–*x*_Fe_2_O_4_ (*x* = 0–1) Nanoparticles in Magnetic Fluid Hyperthermia: Correlation with Cation Distribution and Magnetostructural Properties

**DOI:** 10.1021/acsomega.2c05651

**Published:** 2022-11-22

**Authors:** Satish S. Phalake, Manohar S. Lad, Ketaki V. Kadam, Syed A. M. Tofail, Nanasaheb D. Thorat, Vishwajeet M. Khot

**Affiliations:** † Department of Medical Physics, Centre for Interdisciplinary Research, D. Y. Patil Education Society (Institution Deemed to Be University), Kolhapur416 006, Maharashtra, India; ‡ Department of Physics and Bernal Institute, University of Limerick, Castletroy, Co. Limerick, LimerickV94T9PX, Ireland; § Nuffield Department of Women’s and Reproductive Health, John Radcliffe Hospital, Medical Sciences Division, University of Oxford, OxfordOX3 9DU, U.K.

## Abstract

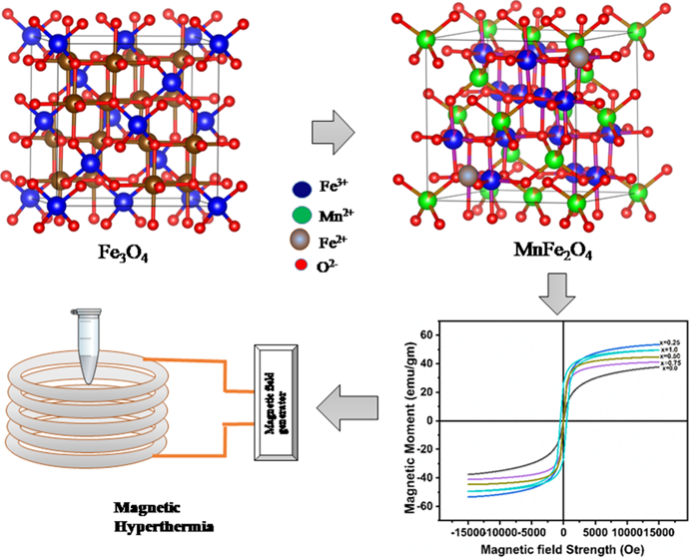

Optimization of manganese-substituted iron oxide nanoferrites having the composition Mn_*x*_Fe_1–*x*_Fe_2_O_4_ (*x* = 0–1) has been achieved by the chemical co-precipitation method. The crystallite size and phase purity were analyzed from X-ray diffraction. With increases in Mn^2+^ concentration, the crystallite size varies from 5.78 to 9.94 nm. Transmission electron microscopy (TEM) analysis depicted particle sizes ranging from 10 ± 0.2 to 13 ± 0.2 nm with increasing Mn^2+^ substitution. The magnetization (*M*_s_) value varies significantly with increasing Mn^2+^ substitution. The variation in the magnetic properties may be attributed to the substitution of Fe^2+^ ions by Mn^2+^ ions inducing a change in the superexchange interaction between the A and B sublattices. The self-heating characteristics of Mn_*x*_Fe_1–*x*_Fe_2_O_4_ (*x* = 0–1) nanoparticles (NPs) in an AC magnetic field are evaluated by specific absorption rate (SAR) and intrinsic loss power, both of which are presented with varying NP composition, NP concentration, and field amplitudes. Mn_0.75_Fe_0.25_Fe_2_O_4_ exhibited superior induction heating properties in terms of a SAR of 153.76 W/g. This superior value of SAR with an optimized Mn^2+^ content is presented in correlation with the cation distribution of Mn^2+^ in the A or B position in the Fe_3_O_4_ structure and enhancement in magnetic saturation. These optimized Mn_0.75_Fe_0.25_Fe_2_O_4_ NPs can be used as a promising candidate for hyperthermia applications.

## Introduction

Due to their unique physical features, known biocompatibility, ease of production, and highly adjustable nature at the nanoscale, maghemite (γ-Fe_2_O_3_) and magnetite (Fe_3_O_4_) nanoparticles (NPs) are especially well suited for various biomedical applications.^1,2^ Magnetization in Fe_3_O_4_ can be tuned by replacing iron ions with transition metal cations, especially manganese ions, which have higher magnetic moments.^3^ It is explored for many applications which include catalysts, humidity sensors, biomedicine, MRI, microwave technologies, drug delivery, and magnetic fluid hyperthermia.^2^ The properties of manganese ferrites such as their high electrical resistance, high curie temperature (bulk MnFe_2_O_4_ is *T*_c_ 577 K), low coercivity value, and a low eddy current loss allow them to serve a wide range of applications.^4,5^ The integration of secondary cations Mn^2+^ in Fe_3_O_4_ and synthesis reproducibility have been studied.^6^ In the past decade, the general term MFe_2_O_4_ (M = Co, Mg, Ni, *etc.*) of spinel ferrites has been widely used for a variety of technological and biomedical applications.^7,8^ The magnetic and electrical properties of these compounds strongly depend on the synthesis process, chemical content, annealing temperature, and cation distribution. The cation distribution in spinel ferrite materials among two interstitial sites of the structure is one of the most challenging aspects of studying these materials due to its effect on the properties of ferrites.^9^ Shahane *et al.* reported the MnFe_2_O_4_ magnetic NPs (MNPs) showing the antiparallel spin moments among Fe^3+^, Mn^2+^, and Fe^2+^ ions at A-sites and inverse spinel structures.^10^ The polycrystalline spinel ferrite Co_*x*_Ni_1–*x*_Fe_2_O_4_ (*x* = 0–1) was obtained by the sol–gel autocombustion method with the Co substitution. In Co^2+^-substituted nickel ferrite, the density is higher than Ni^2+^ ions, owing to the higher magnetocrystalline anisotropy and the smaller particle size. The saturation magnetization (*M*_s_) was increased to *x* = 0.8, at which point there was a small reduction in *M*_s_ for CoFe_2_O_4_.^11^ There have been several proposals for substituted magnetite NPs, M_*x*_Fe_3–*x*_O_4_ (M = Ni, Zn, Mn, and Co, 0 < *x* ≤ 1) for various bio-applications since their magnetic properties can be easily controlled by replacing divalent or trivalent metal ions without modifying the crystal structure by either replacing them completely or partially.

Mn_*x*_Fe_3–*x*_O_4_ NPs among these ferrites show stronger magnetization (*M*_s_), low coercivity (*H*_c_), and low inherent toxicity than other doped ferrite materials and in some cases even higher *M*_s_ than the best studied and currently available iron oxide NPs, along with good chemical stability and biocompatibility. Additionally, manganese ferrites can be modified by tuning hyperthermic therapeutic temperature which is possibly suitable for self-controlled hyperthermia treatment.^8,12^ The formula of a metal ferrite material is most precisely described as (M_*x*_Fe_1–*x*_)[M_*x*_Fe_2–*x*_]O_4_, “A” tetrahedral site and “B” octahedral site are denoted, respectively, by parentheses and square brackets, and *x* is the inversion parameter quantifying the distribution of M^2+^, Fe^2+^, and Fe^3+^ cations among these sites. The manganese substitution was performed in crystals by changing the molar concentration of Mn^2+^, and a variation of Mn_*x*_Fe_1–*x*_Fe_2_O_4_ with a change in molar ratios of Mn^2+^ to Fe^2+^ were synthesized, where “*x*” varies from 0 to 0.75. Manganese ferrites (MnFe_2_O_4_) are presented as one of the most promising materials due to their magnetization capacity.^13^ Yousuf *et al.* synthesized yttrium-substituted manganese ferrites using a reverse-micelle micro-emulsion method and found that with the increase of the yttrium content, the lattice constant increased.^14^ A variety of metal ions in spinel ferrite lead to structural distortions, which affect the material’s physical and structural characteristics, as well as structural parameters.^15^ The distribution of ions between the tetrahedral and octahedral sites, as well as their interaction, ultimately decides the magnetic characteristics of NPs.^16^

There are different methods for the synthesis of ferrite NPs, such as chemical co-precipitation,^3^ a sol–gel autocombustion method,^17^ combustion synthesis,^18^ ultrasonically assisted co-precipitation method,^19^ and thermal decomposition method.^20^ Thermal decomposition and co-precipitation methods are generally used for the synthesis of NPs. In an organic medium, ferrite NPs of a controlled size can be easily obtained using the former method. Chemical co-precipitation is the simplest methods for the synthesis of MNPs in an aqueous solution. By optimizing the synthesis parameters such as concentration, pH, temperature, the size of the NPs can be varied.^10^ Though there is a tremendous advancement in the field of material chemistry, obtaining MNPs with improved magnetic properties and monodisperse nature still poses challenges to the scientific community in this field.

Magnetic fluid hyperthermia is an application of MNPs in cancer and presents a non-invasive treatment option in which NPs at the tumor site raise to a temperature of 42–46 °C. The heating efficacy of MNPs is measured in terms of specific absorption rate (SAR), which largely relies on parameters such as size, shape of NPs, magnetization, strength of the applied field and frequency, and so forth. Cation distribution in ferrites also tends to affect the magnetic properties substantially.^21,22^ The temperature response by MNPs under AC magnetic field is also determined by interparticle interaction, particle concentration in the carrier liquid, viscosity, heat capacity, and surface modification.^23^ In the present work, a systematic evaluation of the substitution of Mn^2+^ into Mn_*x*_Fe_1–*x*_Fe_2_O_4_ (*x* = 0.25, 0.50, 0.75, and 1.0) has been carried out by correlating induction heating studies of MNPs with cation distribution.

## Experimental Section

### Materials

The materials were used to synthesize MNPs: FeCl_3_·6H_2_O, ≥99%; FeCl_2_·4H_2_O, ≥99%; MnCl_2_·4H_2_O, ≥99%; and NaOH, ≥99% purchased from Sigma-Aldrich. All the chemicals were used without further purification and are water soluble.

### Synthesis of Manganese Iron Oxide NPs

In the typical synthesis of Mn_0.25_Fe_0.75_Fe_2_O_4_ (0.25 mmol), manganese(II) chloride, (2 mmol) iron(III) chloride hexahydrate, and (0.75 mmol) iron(II) chloride tetrahydrate were separately dissolved in distilled water with constant stirring. Then, until the occurrence of co-precipitation at pH 12, (8 mmol) sodium hydroxide (NaOH) was added directly to the above solution as a precipitating agent and kept at 70–80 °C for 2 h. The precipitate was collected by magnetic decantation and washed with double-distilled water. The washed precipitate was dried at room temperature overnight. Mn_*x*_Fe_1–*x*_Fe_2_O_4_ (*x* = 0.25, 0.50, 0.75, and 1.0) was prepared using the same procedure.

### Characterization

The X-ray diffraction (XRD) patterns of powder samples with various concentrations were recorded using Cu Kα radiation at the wavelength (λ) = 1.546 Å. The crystallite size of the samples was calculated using the Scherrer equation
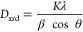
1where “*D*_xrd_” is the crystalline size, “*K*” is the Scherrer constant, β is the FWHM (full width at half-maximum), and θ is the diffraction angle. The formula has been used to derive the lattice constant “*a*” using the calculated corresponding *d* values
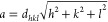
2X-ray density (*d*_x_) of the material
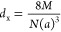
3where “*M*” is the atomic weight and “*N*” is Avogadro’s number (6.022 × 10^23^ mol^–1^).

The Fourier transform infrared (FTIR) spectroscopy study of samples with different concentrations was obtained using an Alpha (II) Bruker unit in the range of 400–4000 cm^–1^. Transmission electron microscopy (2100F JEOL TEM) was employed to observe the size and shape of NPs. Magnetization–field (*M*–*H*) measurements were conducted at room temperature at fields up to 15 kOe using a vibrating sample magnetometer.

An EasyHeat 8310 (Ambrell, UK) was used to study induction heating of the as-prepared MNPs in a physiological medium at an applied fixed frequency of 277 kHz. The field amplitude was adjusted from 13.3 to 26.7 kA/m.

## Results and Discussion

### Structure, Phase, and Morphology Analysis

**Figure 1:**
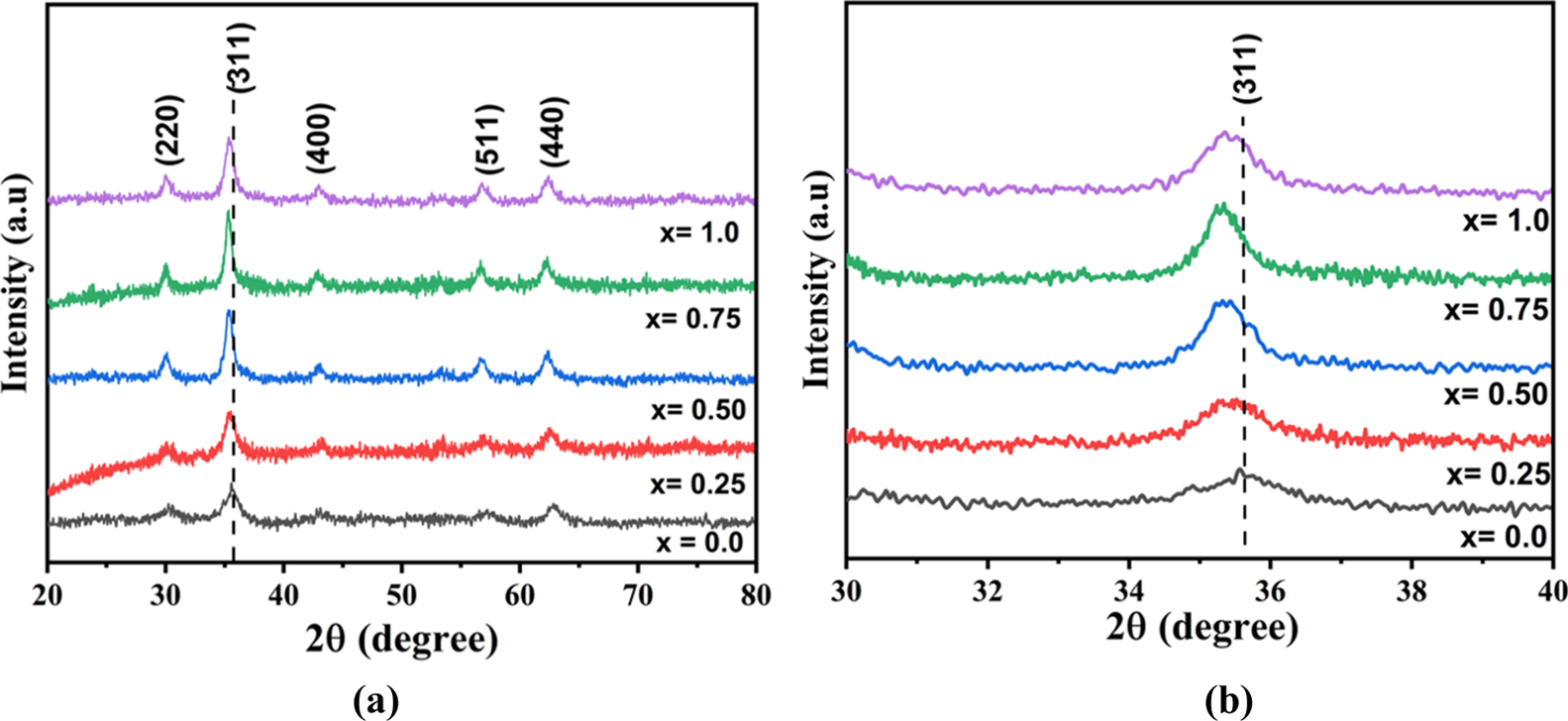
(a) XRD patterns for Mn_*x*_Fe_1–*x*_Fe_2_O_4_ (*x* = 0–1) NPs and (b) shift view of the region around the (311) peak at different Mn^2+^ ions.

The crystallographic structure and crystallite size were determined using the XRD patterns of Mn_*x*_Fe_1–*x*_Fe_2_O_4_ NPs with (*x* = 0–1) in the 2θ range 20–80° and are shown in Figure 1a. The XRD patterns reveal broad peaks and crystallite sizes, and samples are crystalline in nature; their profiles are matched with JCPDS card nos. 00-019-0629 for Fe_3_O_4_ and 00-010-0319 for MnFe_2_O_4_. The XRD results confirm the formation of cubic ferrite with the space group *Fd*3*m*. An enlarged view of the high-intensity characteristic peak (311) shows a shift to lower angles with increasing Mn^2+^ substitution (Figure 1b). It is due to the expansion of the unit cell as Mn^2+^ ions are substituted in the magnetite structure.^3^ The increase in lattice constant (*a*) with an increase in Mn^2+^ is explained using ionic radii, where the radius of Mn^2+^ (0.80 Å) is larger than that of Fe^2+^ (0.77 Å) and Fe^3+^ (0.64 Å), causing lattice expansion; as a result, the lattice parameter increases from 0.8350 to 0.8409 nm due to unit cell dimension expansion.^24^

The inverse spinel manganese iron oxide will eventually expand when small-sized Fe^3+^ and Fe^2+^ ions are replaced with large-sized Mn^2+^ ions. The strain on an inverse spinel-type structure will be linear in the lattice because of the elastic deformation caused by substituting Mn^2+^ ions. This effect is because the spacing in the lattice plane changes and the peaks shift to lower 2θ positions (Figure 1b).^25^ The calculated lattice parameter “*a*” with different compositions is shown in Table S1 (Supporting Information).

**Figure 2:**
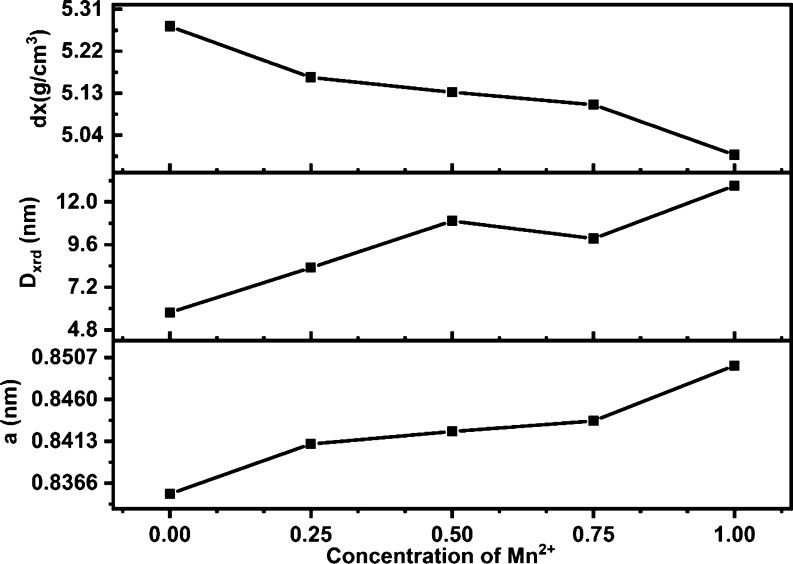
Variation of the Mn_*x*_Fe_1–*x*_Fe_2_O_4_ (*x* = 0–1) NPs with Mn^2+^ content *x* in terms of their lattice parameter *a* (nm), crystallite size *D*_xrd_ (nm), and X-ray density *d*_x_ (g/cm^3^).

The calculated crystallite size (*D*_xrd_) of synthesized manganese iron oxide nanocrystals varies from 5.7 to 12.92 nm with varying Mn^2+^ concentrations. The calculated unit cell volume increases from 0.583 to 0.613 nm^3^, and the equivalent values of X-ray density (*d*_x_) decrease from 5.27 to 4.99 g/cm^3^ with the increase in Mn^2+^, respectively (Figure 2).^26,27^

**Figure 3:**
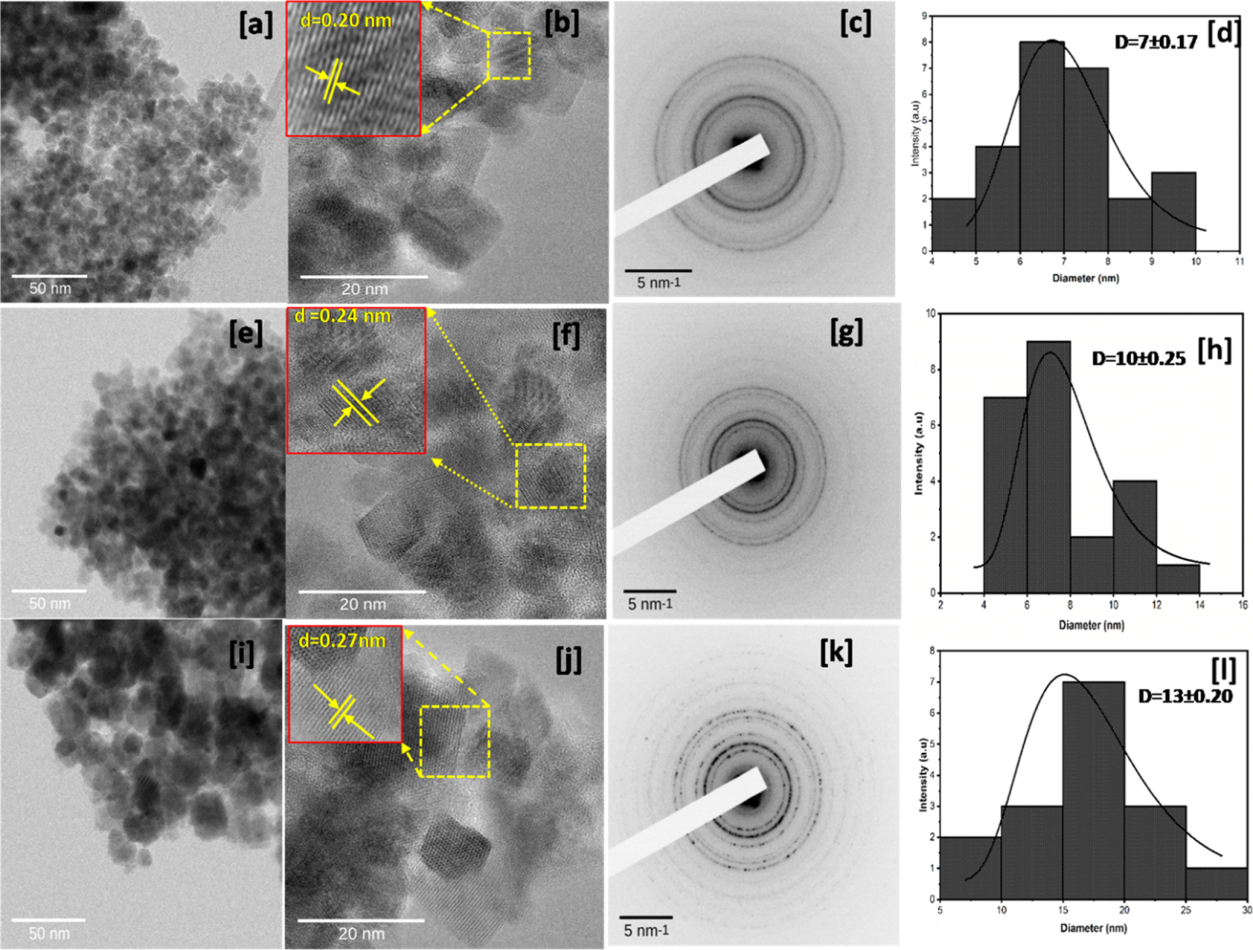
Images of (a–d), (e–h), and (i–l) represent the TEM images, SAED patterns, and histograms of samples Mn_*x*_Fe_1–*x*_Fe_2_O_4_ at *x* = 0, 0.25, and 0.75, respectively.

Figure 3a,e,i shows the TEM images of manganese iron oxide NPs, and the corresponding histograms illustrate particle size distribution for three different compositions. The TEM analysis for samples Mn_*x*_Fe_1–*x*_Fe_2_O_4_ (*x* = 0, 0.25, and 0.75) shows the particle size and distribution, which is in good agreement with those determined by XRD. The product contains agglomerated NPs with spherical and cubic forms, as can be seen in Figure 3. Also, (c,g,k) depict the equivalent selected area electron diffraction (SAED) patterns of NPs. The picture shows spotty ring patterns, which are consistent with the XRD results, indicating that NPs have a good crystal structure. Figure 3b,f,j depicts the lattice fringes confirming the single nature of the core, with a lattice spacing of roughly 0.20–0.27 nm, which corresponds to the (311) lattice plane.^28^Figure 3d,h,l, provides the histogram, and formation of NPs with sizes around 7 ± 0.17 to 13 ± 0.2 nm is confirmed.

The chemical composition of the obtained Mn_*x*_Fe_1–*x*_Fe_2_O_4_ (*x* = 0–1) NPs was studied using energy-dispersive X-ray (EDX) analysis (shown in Figure S1, Supporting Information). The analyzed results, as shown in the respective Figure S1, confirm the percentage of Mn, Fe, and O elements which are presented in Table1. The phase purity of samples confirms that samples conform to the expected composition ratio and confirms that stoichiometry is properly maintained during preparation, implying that the expected ratio is maintained during preparation.

**Table 1:** Stoichiometry % Concentration of the Constituent Elements of the Mn_*x*_Fe_1–*x*_Fe_2_O_4_ (*x* = 0–1) NPs by EDX

sample Mn_*x*_Fe_1–*x*_Fe_2_O_4_ (*x*)	Mn	Fe	O
0.0	0.0	27.30	73.70
0.25	2.91	25.50	71.60
0.50	6.21	35.75	58.04
0.75	9.02	25.07	65.90
1.00	10.75	21.79	67.46

### Cation Distribution

Nanomaterials present novel properties as compared to their bulk counterparts; strains on the surface or interface are most of the important basic quantities to a wide variety of domains.^9,29^ During compression or tension, nanoscale materials can modify their lattice parameters, thereby changing their intrinsic bond distances and electron energy levels. Calculations of the grain size and micro-strain created throughout the process were made using the line width FWHM (in radians) of the powder XRD lines. As a result of equation β, the width of the integral line is given by
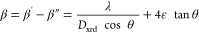
4where β′ and β″ are the contributions of grain size and strain, respectively, θ is the Bragg angle, ε is the strain, and “*D*_xrd_” is the crystallite size. When the strain term β″ = 4ε tan θ is negligible, ε can be evaluated in terms of β. For various XRD lines corresponding to different planes, the integral line width is measured, and eq 4 can be simplified as
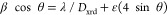
5The values of β cos θ and 4 sin θ have a linear relationship. The strain (ε) evaluated from the intercept = λ/*D*_xrd_ on the *y*-axis when plotting β cos θ (*y*-axis) *versus* 4 sin θ (*x*-axis).

**Figure 4:**
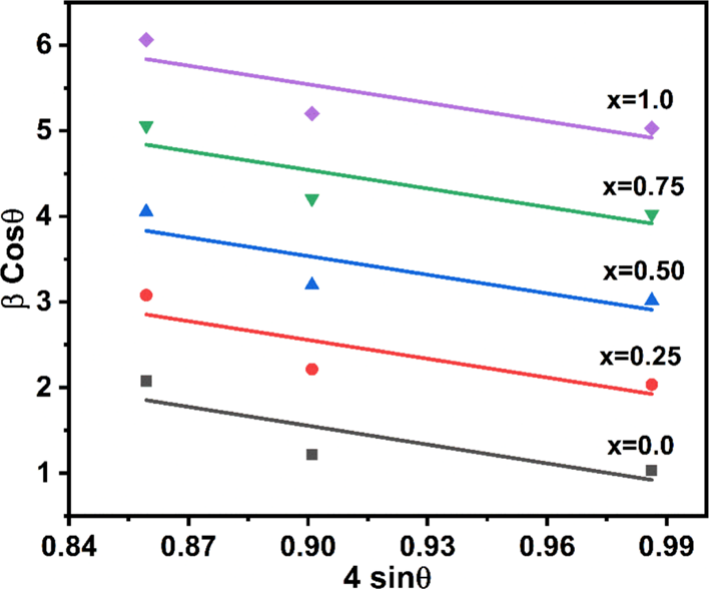
Strain graph of Mn_*x*_Fe_1–*x*_Fe_2_O_4_ (*x* = 0–1) NPs.

The strain measurements for each sample are shown in Figure 4. For all the sample, the linear difference of 4 sin θ with β cos θ can be seen. Strain measurement from the slope is more sensitive to increased Mn^2+^ content *x*, indicating that a larger amount of Mn^2+^ can be accommodated in the matrix of Mn_*x*_Fe_1–*x*_Fe_2_O_4_. Differences in ionic size between the two cations account for the difference in cation distribution on tetrahedral and octahedral sites.^30^

The spinel ferrites having structural and magnetic properties are affected by cation distribution in crystal lattices. An inverse phase cubic spinel structure has been observed for manganese iron oxide nanocrystals, with Fe^2+^ ions occupying B-sites and Mn^2+^, Fe^3+^ ions equally distributed in the A- and B-sites. Studies of cation dispersion in spinel ferrite give useful information for improving materials with desirable characteristics.^31^ In Mn_*x*_Fe_(1–*x*)_Fe_2_O_4_, XRD analysis was used to determine the distribution of cations Mn^2+^, Fe^2+^, and Fe^3+^ among octahedral and tetrahedral sites. The cation distribution in spinel ferrite was determined by comparing experimentally measured diffraction intensities with those calculated for a wide number of hypothetical crystal forms. Various distribution parameters are used to calculate the intensity using the Burger formula for the planes^32^
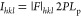
6*F* is the structural component, *P* is the multiplicity, and *L*_p_ is the Lorentz polarization factor in this equation.
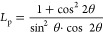
7

The best information on cation distribution is obtained by comparing experimental and estimated intensity ratios for reflections whose intensities (i) are relatively independent of the oxygen parameter, (ii) change with the cation distribution in different ways, and (iii) do not differ significantly. Fe^3+^ ions have no preference for the lattice site and can occupy any of the two; Mn^2+^ ions can likewise occupy both sites. Mn^2+^ and Fe^3+^ ions have a strong A-site preference in Mn_*x*_Fe_1–*x*_Fe_2_O_4_, while Fe^2+^ and Fe^3+^ ions occupy the B-sites.^32^ The following cation distribution can be proposed since Mn_*x*_Fe_1–*x*_Fe_2_O_4_ (*x* = 0–1) form inverse spinel
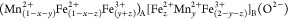
8where *y* and *z* are the Mn^2+^ and Fe^2+^ ion concentrations at their respective sites and 0 ≤ *x* ≤ 1. The following equation and an acceptable cation distribution are used to determine the mean ionic radii of tetrahedral (A) and octahedral (B) sites (*r*_A_ and *r*_B_)
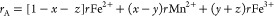
9
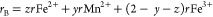
10Using the value of *a*, the radius of oxygen ion *R*_o_ = 1.32 Å, and, *r*_A_, the oxygen positional parameter (*u*) can be obtained as follows
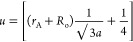
11

With the increasing Mn^2+^ content *x*, it is apparent that *r*_B_ decreases and *r*_A_ increases. The relative value of Mn^2+^, Fe^2+^, and Fe^3+^ occupancy with their different ionic radii in the tetrahedral site helps to explain the difference in calculated tetrahedral or octahedral radius. The oxygen positional parameter “*u*” rises to 0.401 from 0.382. If “*u*” = 3/8 = 0.375 in an ideal fcc structure, the “*u*” values of most ferrites are greater than this ideal value, indicating that the oxygen ions are transferred in such a way in the A–B interaction that the distance between A and O ions increases, while the distance between B and O ions decreases. As a result, the A–A interaction decreases, while the B–B interaction increases.^9,18^

Using the estimated values of *r*_A_ and *r*_B_, the theoretical lattice parameter (*a*_th_) is determined as
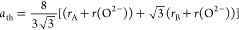
12

Theoretical lattice constant values are shown in Table 2 for Mn_*x*_Fe_1–*x*_Fe_2_O_4_ (*x* = 0–1) nanocrystals.

**Table 2:** Distribution of Cations among A- and B-Sites, ionic Radii of tetrahedral (rA) and octahedra sites (rB), oxygen parameter (u), theoretical lattice parameter (ath) and strain (ε)

comp	A-site			B-site							
*x*	Mn^2+^	Fe^2+^	Fe^3+^	Mn^2+^	Fe^2+^	Fe^3+^	*r*_A_ (Å)	*r*_B_ (Å)	“*u*” (Å)	*a*_th_	strain
0.0	0.0	0.05	0.95	0.0	0.95	1.05	0.59	1.58	0.382	0.8352	0.7229
0.25	0.12	0.0	0.88	0.13	0.75	1.12	0.66	1.51	0.385	0.8405	0.7249
0.50	0.19	0.0	0.81	0.31	0.50	1.19	0.74	1.45	0.391	0.8423	0.7258
0.75	0.25	0.0	0.75	0.50	0.25	1.25	0.82	1.39	0.396	0.8435	0.7309
1.00	0.29	0.0	0.71	0.71	0.0	1.29	0.91	1.34	0.401	0.8497	0.7333

As the concentration of Mn^2+^ increases, the X-ray density rises linearly because the iron atom is lighter than the manganese atom. The distance between magnetic ions is calculated in the tetrahedral site *L*_A_ and octahedral site *L*_B_.
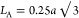
13
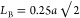
14Table 3 shows the calculated values for *L*_A_ and *L*_B_. It is observed that with the increase in the Mn^2+^ content, the hopping length also increases.

**Table 3:** Hopping Length (*L*_A_) and (*L*_B_), Tetrahedral Bond Length (*d*_Ax_), Octahedral Bond Length (*d*_Bx_), Tetrahedral Edge (*d*_AxE_), and Shared (*d*_BxE_) and Unshared (*d*_BxEu_) Octahedral Edges as a Function of *x* for Mn_*x*_Fe_1–*x*_Fe_2_O_4_ (*x* = 0–1) NPs

sample Mn_*x*_Fe_1–*x*_Fe_2_O_4_ (*x*)	*L*_A_ (nm)	*L*_B_ (nm)	*d*_Ax_ (nm)	*d*_Bx_ (nm)	*d*_AxE_ (nm)	*d*_BxE_ (nm)	*d*_BxEu_ (nm)	α	β
0.0	0.3617	0.2953	0.1894	0.2037	0.3095	0.2811	0.2954	0.4108	0.3871
0.25	0.3641	0.2973	0.1906	0.2051	0.3115	0.2830	0.2973	0.4155	0.2931
0.50	0.3647	0.2978	0.1910	0.2054	0.3121	0.2834	0.2979	0.4051	0.3908
0.75	0.3652	0.2982	0.1913	0.2057	0.3125	0.2838	0.2983	0.4117	0.1846
1.00	0.3679	0.3004	0.1925	0.2072	0.3148	0.2859	0.3005	0.4074	0.3426

According to eqs 15 and 16, one can calculate the shortest distance between A-site cations and oxygen ions and that between B-site cations and oxygen ions, respectively.
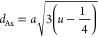
15
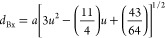
16Equations 17–19 were used to determine the A-site edge “*d*_AxE_”, the shared B-site edge “*d*_BxE_”, and the unshared B-site edge “*d*_BxEu_”.
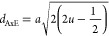
17
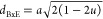
18
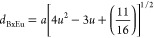
19

As shown in Table 3, substitution with Mn^2+^ indicates an increase in the octahedral bond distance *d*_Bx_ and the tetrahedral bond distance *d*_Ax_. In manganese iron oxide nanocrystals, due to the extension of octahedral B-sites, differences between *d*_AxE_ and *d*_BxE_ increase because of the larger radius of Mn^2+^ ions compared to Fe^2+^ and Fe^3+^ ions. As a result, the oxygen anions are displaced relative to each other, causing the tetrahedral A-sites to decrease. Because there is more covalent bonding at the A-sites than at the B-sites as a result of shrinkage, the force constant between the cations and anions increases. There is an increase in the value of tetrahedral edge “*d*_AxE_”, shared octahedral edge “*d*_BxE_”, and unshared octahedral edge “*d*_BxEu_” due to Mn^2+^ substitution, which is shown in Table 3. These modifications are due to Mn^2+^ greater ionic radius, which cause the octahedral site to expand while the tetrahedral site shrinks.^33−35^

In terms of *r*_A_, *r*_B,_*a*_th_, *a*_exp_, and *r*(O^2–^), the following relationships can be used to describe the degree of ionic packing (α) and the vacancy parameter (β).
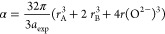
20
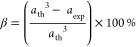
21

The vacancy parameter reveals the presence of vacancies at both tetrahedral and octahedral sites and offers the normalized volume of the missing ions, which is a total measure of vacancy concentration in the spinel structure.^36^ The magnetic properties of spinel ferrite nanocrystals are attributed to octahedral and tetrahedral sites, as well as their relative strengths, which are affected by magnetic ion accumulation on the surfaces of these crystals’ inter-lattice and inter-sublattice interactions. Variations in the number of magnetic ions in both sites alter the magnetic properties. Ferrite nanocrystals substituted with Mn^2+^ expand the tetrahedral site resulting in an increase in the bond distance at the A-site. The structural features of ferrites are mostly influenced by the variation in bond distance between a cation and cation, as well as a cation and an anion, at various magnetic parameters.^37^

As the unit cell volume increases, all values of inter-ionic distances increase. This is because the smaller ionic radii Fe^2+^ is replaced by a more radially large Mn^2+^, which has a smaller interionic distance between ions (*b*, *c*, *d*, *e*, *f*) for Mn_*x*_Fe_1–*x*_Fe_2_O_4_ (*x* = 0–1) nanocrystals. From Tables 4 and 5, it is observed that an increase in cation–anion length and cation–cation length with Mn^2+^ substituted resulted in a decreased superexchange strength compared to iron oxides.^38^ The inter-ionic lengths and angles between the cation–anion and cation–cation play a major and effective influence in magnetic interactions. Different configurations of the ion pairs with favorable angles for the individual magnetic interactions and inter-ionic distances give the cation–anion distances *p*, *q*, *r*, and *s*, as well as the cation–cation distances (*b*, *c*, *d*, *e*, and *f*) and the respective bond angles θ_1_, θ_2_, θ_3_, θ_4_, and θ_5_. The inter-ionic distances are determined by the crystalline structure and magnetic characteristics.^36^

**Table 4:** Calculation of Distances between Cations and Anions and between Cations and Cations for Mn_*x*_Fe_1–*x*_Fe_2_O_4_ (*x* = 0–1) Nanocrystals

sample Mn_*x*_Fe_1–*x*_Fe_2_O_4_ (*x*)	*p* (nm)	*q* (nm)	*r* (nm)	*s* (nm)	*b* (nm)	*c* (nm)	*d* (nm)	*e* (nm)	*f* (nm)
0.0	0.20	0.1808	0.3462	0.5512	0.2953	0.3462	0.3617	0.5426	0.5115
0.25	0.2102	0.1820	0.3486	0.5549	0.2973	0.3486	0.3641	0.5462	0.5149
0.50	0.2106	0.1823	0.3492	0.5559	0.2978	0.3492	0.3647	0.5471	0.5158
0.75	0.2109	0.1826	0.3496	0.5566	0.2982	0.3496	0.3652	0.5479	0.5165
1.00	0.2124	0.1839	0.3522	0.5607	0.3004	0.3522	0.3679	0.5519	0.5203

**Table 5:** Calculated Values of Hopping Lengths and Inter-ionic Bond Angles for Mn_*x*_Fe_1–*x*_Fe_2_O_4_ (*x* = 0–1) Nanocrystals

sample Mn_*x*_Fe_1–*x*_Fe_2_O_4_ (*x*)	*L*_A_ (nm)	*L*_B_ (nm)	θ_1_	θ_2_	θ_3_	θ_4_	θ_5_
0.0	0.3617	0.2953	125.24	154.93	90.00	68.11	79.99
0.25	0.3641	0.2973	125.30	154.82	90.01	68.10	79.98
0.50	0.3647	0.2978	125.28	154.74	89.98	68.10	79.97
0.75	0.3652	0.2982	123.20	154.86	89.97	68.11	79.99
1.00	0.3679	0.3004	123.27	154.85	90.00	68.11	79.99

The following equations are used to calculate these values, as shown in Table 4 taking into account the experimental value of the lattice constant and oxygen parameters.
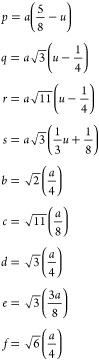
22As a result of considering the following equations with the inter-ionic lengths measured, we can obtain the bond angles for manganese iron oxide, and these values are given in Table 5.
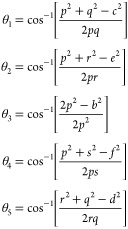
23

Because these angles are related to A–B and A–A interactions, a rise in these angles verifies the strength of these bonds as bond length and bond angle both increase with the substitution of Mn^2+^.

### FTIR Analysis

**Figure 5:**
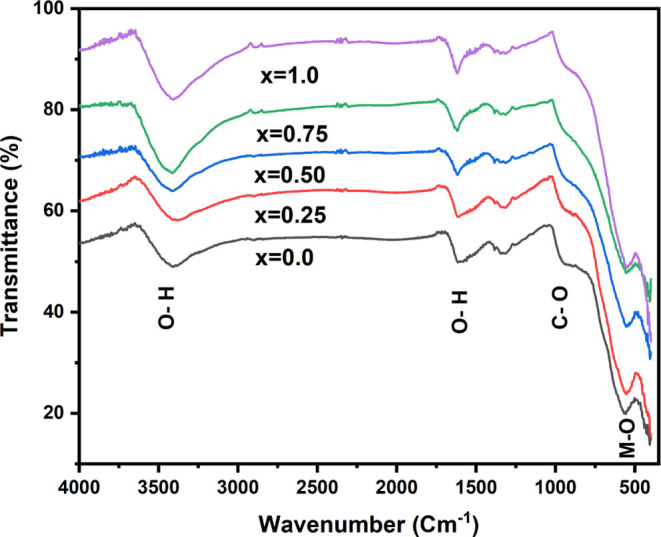
FTIR spectra of Mn_*x*_Fe_1–*x*_Fe_2_O_4_ (*x* = 0–1) NPs.

Figure 5 shows the FTIR absorption spectra of the Mn_*x*_Fe_1–*x*_Fe_2_O_4_ (*x* = 0–1) NPs in the range of 4000–400 cm^–1^. The formation of the spinel ferrite phase has been confirmed by an FTIR analysis. There are distinct intensity bands in the FTIR spectra corresponding to the covalent linkages between NPs, such as the M_T_–O–M_O_ stretching band at ∼600–500 cm^–1^, where M_T_ and M_O_ represent the tetrahedral and octahedral sites. The band positions of the synthesized Mn^2+^-substituted nanoferrites are given in Table 6.

**Table 6:** Tetrahedral Band (υ_1_), Octahedral Band (υ_2_), and Force Constants (*f*_T_ and *f*_O_) of Mn_*x*_Fe_1–*x*_Fe_2_O_4_ (*x* = 0–1) NPs

Mn_*x*_Fe_1–*x*_Fe_2_O_4_ (*x*)	υ_1_ (cm^–1^)	υ_2_ (cm^–1^)	*f*_T_ × 10^5^(dyne/cm^2^)	*f*_O_ × 10^5^(dyne/cm^2^)
0.00	557.32	439.52	2.2614	1.4075
0.25	556.36	433.90	2.2536	1.3707
0.50	554.43	427.15	2.2380	1.3284
0.75	555.39	426.19	2.2458	1.3224
1.00	553.47	425.27	2.2303	1.3167

As can be seen, the characteristic band of M^2+^–O has decreased from a value of 557.37–553.47 cm^–1^ at tetrahedral sites and 439.52–425.27 cm^–1^ at octahedral sites with increasing Mn^2+^ concentration. The bands around 1617 and 3412 cm^–1^ are attributed to the bending vibrational modes of the adsorbed water molecules.^39^

The Fe^3+^–O^2–^ stretching vibrations change when Fe^2+^ ions at both sites in the ferrite lattice are substituted by Mn^2+^ ions with a large ionic radius and atomic weight. The FTIR provides information regarding the variation in the molecular structure of ferrite resulting from the addition of Mn^2+^ ions to Fe^3+^–O^2–^.^18^ The band shift of υ_1_ and υ_2_ to a lower frequency reveals variation in *f*_T_ and *f*_O_ for the A and B-sites because the vibration frequency (υ) is proportional to the force constant “*f*” as
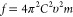
24where “*m*” is the reduced mass for the Fe^3+^ ions and the O^2–^ ions (2.065 × 10^–23^ g/mol) and *C* is the speed of light.

By using far-infrared absorption, the cation distribution can be studied since cations in the system at both A- and B-sites are sensitive to changes in the system. In crystalline solids, it is also possible to determine local symmetry surface defects, oxidation, and phenomenon associated with the spinel structure, as well as the presence or absence of Fe^2+^ ions.^23^

### Magnetic Properties

**Figure 6:**
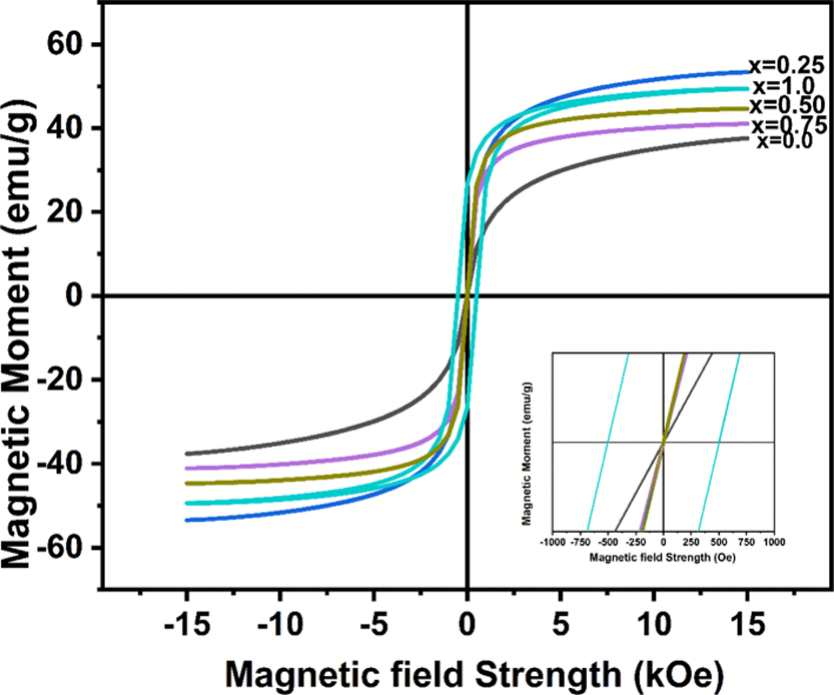
Magnetization (*M*) *vs* field (*H*) curves of the Mn_*x*_Fe_1–*x*_Fe_2_O_4_ (*x* = 0–1) NPs.

A magnetic field of 15 kOe was applied to the as-prepared samples, giving rise to magnetic hysteresis loops at room temperature (Figure 6). In Table 7, the magnetic properties of the Mn_*x*_Fe_1–*x*_Fe_2_O_4_ (*x* = 0–1) NPs are presented. The net magnetization (*M*_s_) values were found to be 37.63, 53.42, 49.45, 41.06, and 44.65 emu/g for Mn_*x*_Fe_1–*x*_Fe_2_O_4_ (*x* = 0, 0.25, 0.50, 0.75, and 1.0), respectively. Compared to the smaller iron oxide particles that resulted in a higher saturation magnetization, the observed results of the magnetization experiments for Mn_*x*_Fe_1–*x*_Fe_2_O_4_ (*x* = 0–1) are slightly distorted. It could be assigned to the smaller NPs formed during the synthesis and its anisotropic structural composition.^22^ The variation in magnetic properties can also be understood as the distribution of cations among a tetrahedral and octahedral site of spinel ferrite.

**Table 7:** Magnetization (*M*_s_), Remanence (*M*_r_) and Remanence Ratio (*M*_r_/*M*_s_), and Magnetic Moment (*n*_B_) of the Mn_*x*_Fe_1–*x*_Fe_2_O_4_ (*x* = 0–1) NPs

sample *X*	*M*_s_(emu/g)	*M*_r_(emu/g)	*H*_c_ (Oe)	*M*_r_/*M*_s_	*n*_B experimental._	*n*_B calculated_
0.0	37.63	0.09	4.32	0.0023	1.5599	4.1
0.25	53.42	0.44	8.92	0.0082	2.2121	4.25
0.50	49.45	0.15	3.12	0.003	2.0450	4.50
0.75	41.06	0.39	8.37	0.0094	1.6968	4.75
1.0	44.65	0.65	12.11	0.0145	1.8465	5.0

For the series Mn_*x*_Fe_1–*x*_Fe_2_O_4_, the difference of magnetic moment (*n*_B_) with different *x* is calculated. The magnetic moment (*n*_B_) per unit was derived using the following formula and shown in Table 7.
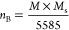
25Here, *M* represents the molecular weight. The magnetic moment values reveal that all of the samples are ferrimagnetic. The magnetic moment of individual ions is calculated using the cation distribution. The cation distribution among A-sites and B-sites affects magnetization. Due to the anti-ferromagnetic coupling between the two sides, a net magnetic moment at zero Kelvin is simply the change in the sublattice magnetizations. The calculation is based on Neel’s two-sublattice model of ferrimagnetism, and the magnetic moment is expressed as the magnetic moment per formula unit in B
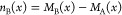
26*M*_B_ and *M*_A_ are the magnetic moments of the B- and A-sites, respectively. Mn_*x*_Fe_1–*x*_Fe_2_O_4_ has a cation distribution where the A-site has a -lower Mn^2+^ ion concentration than the B-site, a mixed spinel structure. Magnetization can be enhanced by the combination of magnetic Mn^2+^ into the B sublattices instead of magnetic Fe^2+^ in the spinel. In the B-site, the magnetic moment is greater than that of the A-site when Mn^2+^ is incorporated. Furthermore, the occupied A-site by the Mn^2+^ ion allows Fe^3+^ ions to transfer from the A-site to the B-site, which in turn increases the total magnetic moment. The findings show that as the Mn^2+^ concentration *x* increases, both the observed and computed values of magneton number increase. When the content of Mn^2+^ is increased to oppose the growth of *M*_s_, coercivity “*H*_c_” increases. This is consistent with the relations *H*_c_ ∝ *K*(μ_0_*M*_s_)^−1^, where μ_0_ is the permeability of free space and *K* is the anisotropy constant.^40^ However, iron oxide NPs are frequently reported to have low magnetization than the bulk phase due to the canting of the spins at the surface and or in the core, which is brought on by decreased coordination and broken super-exchange bonds.^41^ The Mn^2+^-substituted iron oxide crystals have a small variation in the saturation magnetization of nanocrystals. However, it was shown that the saturation magnetization had significantly improved with further Mn^2+^ substitution. We also observe that the variation in Mn^2+^ concentration affects the hysteresis curve’s form. When compared to the somewhat bigger iron oxide particles leading to less saturation magnetization, the observed results of the magnetization studies for the varied *x* values were slightly altered. It is possible that this is caused by the particles’ reduced size and structural anisotropy.^42^ High manganese doping levels may cause lattice distortion in manganese ferrite NPs, which may result in poor saturation magnetization, according to analysis of XRD patterns and lattice distances.^43^

### Induction Heating Study

The effect of Mn^2*+*^-substituted Mn_*x*_Fe_1–*x*_Fe_2_O_4_ (*x* = 0–1) for hyperthermia application is explored by correlating their magnetostructural properties to induction heating. Induction heating studies of manganese-substituted iron oxide NPs had not been reported in correlation with the distribution of cations, which significantly affect its magnetic properties. The heating power of the MNPs is measured in SAR, which is an important parameter in magnetic fluid hyperthermia since it quantifies the degree to which the fluid is capable of converting magnetic energy into heat.^44,45^ The growth in temperature *versus* time for samples at different field amplitudes is shown in Figure S2 (Supporting Information). MNPs dissipate heat in AC magnetic fields in the form of SAR (W/g) and ILP (intrinsic loss power), which are calculated by
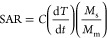
27
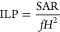
28where *M*_m_ is the magnetic material in suspension, *M*_s_ is the mass of suspension, d*T*/d*t* is the initial slope of the temperature *versus* time graph, *C* is the specific heat capacity of suspension = 4.186 J/(g·°C), *H* is the applied field, and *f* is the frequency. To reduce the amount of magnetic material needed to treat hyperthermia, the SAR value should be as high as possible because it inversely relates to *M*_m_. In total power loss by MNPs in an AC magnetic field, three components are involved: hysteresis loss, eddy current loss, and residual loss.^43^

In an AC magnetic field, the hysteresis loss can be represented as
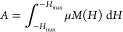
29Thus, the SAR is calculated as
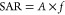
30As a result, one must consider how frequency and amplitude affect SAR. It has also been shown that a human-tolerated range of frequency and amplitude is believed to occur with their product *f* × *H* = *C* not exceeding ∼5 × 10^9^ A/m·s.^46^ The calculated values of *C* are 3.5 × 10^9^, 5.34 × 10^9^, and 7.12 × 10^9^ A/m·s for 13.3, 20, and 26.7 kA/m, respectively. Therefore, in this instance, the essential condition of magnetic field amplitude and frequency is satisfied. Because magnetic field strength and frequency are tightly correlated, SAR values cannot be compared to those of other systems. Thus, discussing heat dissipation in terms of ILP is more appropriate.^47^ Giri *et al.* synthesized Fe_1–*x*_Mn_*x*_Fe_2_O_4_ NPs by the co-precipitation method with a mean size of 10–12 nm, and calorimetric measurements were used to determine the heating efficiency in a field with *f* = 300 kHz and *H* = 10–45 kA/m. The *M*_s_ and SAR of the material had the maximum values for *x* = 0.4 as 85 emu/g and 30 W/g, respectively.^48^ Otero-Lorenzo *et al.* synthesized manganese-doped iron oxide NPs with *M*_s_ values of 66 emu/g and SAR values of 73 W/g of Fe + Mn at *f* = 183 kHz and *H* = 17 kA/m.^49^

**Figure 7:**
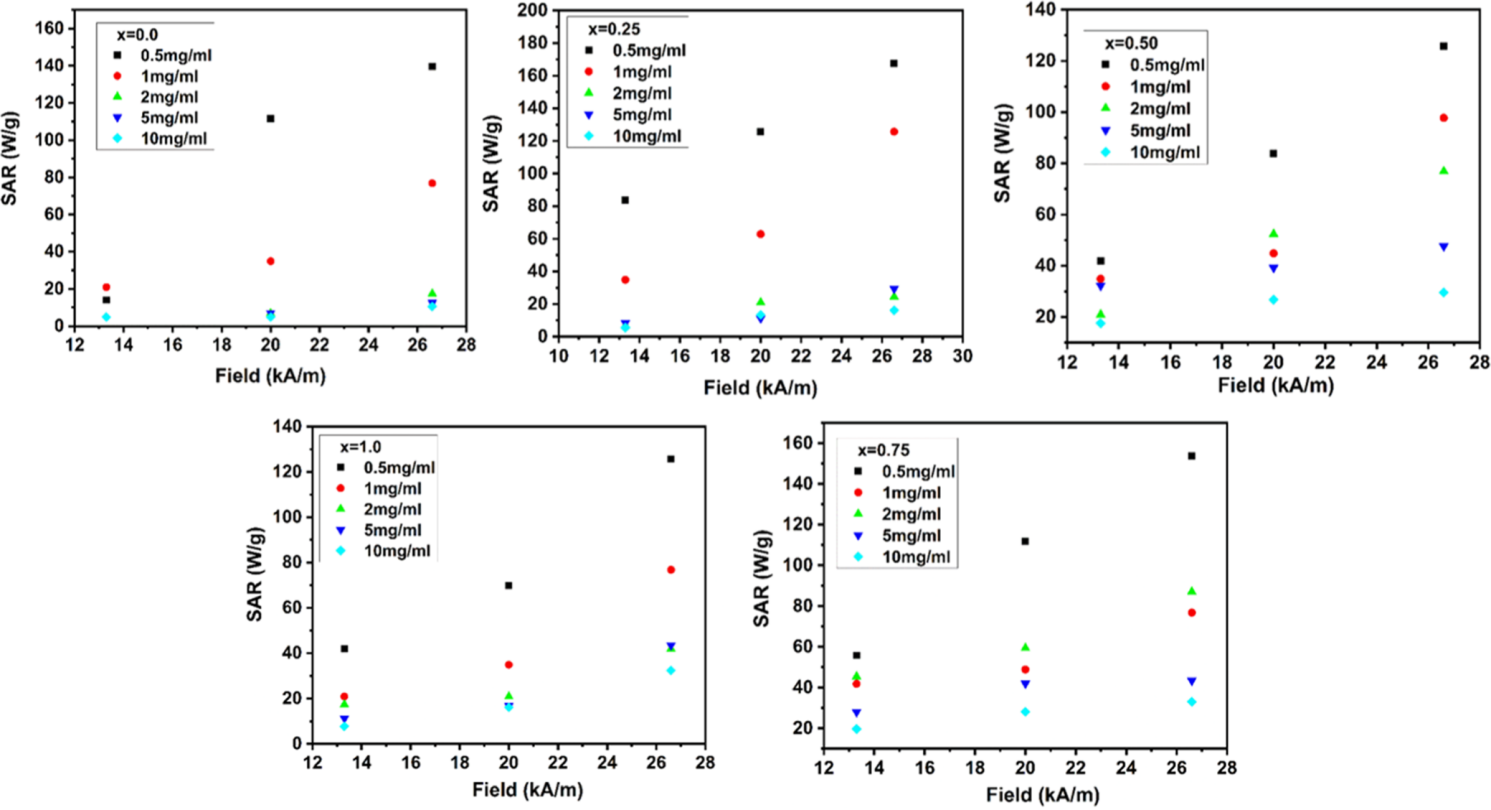
SAR value of the (*x* = 0–1) NPs at different concentrations (0.5, 1, 2, 5, and 10 mg/mL) and applied fields, with constant frequency (277 kHz).

Manganese iron oxide exhibits low conductivity (∼10^–3^ S/cm), which ensures the absence of significant losses from eddy currents and hysteresis when subjected to an external magnetic field.^50^ Therefore, a maximum heat loss in the case of manganese iron oxide may be due to Neel rotation and Brownian losses. The size distribution of magnetic NPs has a significant impact on heat dissipation in an AC magnetic field. The temperature rises with increasing field amplitude and NP concentration (shown in Supporting Information Figure S2). Usually, for hyperthermia therapy, the 42–44 °C temperature is considered as effective.^51^ At concentrations of 5 and 10 mg/mL in water, these NPs self-heated at temperatures rising to 50.25 and 73.32 °C at different magnetic field amplitudes. The actual increase in temperature within 10 min for all samples is measured at a fixed frequency (277 kHz) and different NP concentration (0.5, 1, 2, 5, and 10 mg/mL) at changing magnetic field, 13.3, 20.0, and 26.7 kA/m (Figure S2, Supporting Information) (Figure 7).

Dipole–dipole interactions are affected by broad particle size distributions, affecting the induction heating properties of the material. Consequently, there is an increased hysteresis loss and greater AC magnetically induced heating characteristic. Lasheras *et al.* studied size dependence magnetic hyperthermia of manganese-doped ferrite NPs; it is observed that the SAR is 50–90 W/g and the ILP values are within 1–2 nHm^2^/kg.^26^ Otero-Lorenzo *et al.* synthesized Mn_0.3_Fe_2.7_O_4_ NPS with a solvothermal technique and found the SAR of 37 W/g, while the calculated ILP for the 5 nm particles is 4 nHm^2^/kg.^49^ SAR and ILP values of Mn_*x*_Fe_1–*x*_Fe_2_O_4_ (*x* = 0–1) NPs are calculated from eq 27 and eq 28; shown in Table S4 (Supporting Information) with an increase in -field amplitude from 13.3 to 26.7 kA/m for 0.5, 1, 2, 5, and 10 mg/mL, respectively. The SAR value for Fe_3_O_4_ rises from 4.9 to 17.47 W/g (ILP = 0.09–0.15 nHm^2^/kg) with an increase in field amplitude from 13.3 to 26.7 kA/m for 2 mg/mL, respectively. For sample *x* = 0.25, the value of SAR increases from 5.63 to 29.44 W/g (ILP = 0.41–0.59 nHm^2^/kg) with an increase in the field from 13.3 to 26.7 kA/m. The sample *x* = 0.50 the value of SAR increases from 17.61 to 76.89 W/g (ILP = 0.26–1.68 nHm^2^/kg) with an increase in the field from 13.3 to 26.7 kA/m. The SAR value increases from 19.73 to 87.12 W/g (ILP = 0.67–3.65 nHm^2^/kg) for sample *x* = 0.75 with a field increase from 13.3 to 26.7 kA/m. The SAR increases from 7.75 to 41.94 W/g (ILP = 0.29–1.40 nHm^2^/kg) for sample MnFe_2_O_4_, with a field increase from 13.3 to 26.7 kA/m. The Mn_*x*_Fe_1–*x*_Fe_2_O_4_ (*x* = 0–1) NPs exhibited the highest SAR of about 153.76 W/g for the sample *x* = 0.75 at a physiological safe range of frequency and amplitude. The magnetic field frequency and magnitude determine the ILP parameter, its ILP is from 2 to 4 nHm^2^/kg, the most suitable model, since it can be easily compared across experiments.^52^ When manganese was introduced into the network, local heating increased significantly, from 0.15 nHm^2^/kg (Fe_3_O_4_) to 1.40 nHm^2^/kg (MnFe_2_O_4_). The addition of Mn^2+^, on the other hand, increases the material’s heat which in turn resulted due to improved magnetic properties due to the distribution of cations among A- and B-sites. In the context of a biological application, hyperthermia could damage cancerous cells and protect healthy cells at the same time, while keeping the temperature rise under control as the AMF exposure period increases. In the end, the results clearly show that high values of SAR are not a result of increasing particle concentration. Comparing these values to those reported in the literature, to reach hyperthermia, we used a low concentration and low field.^28^

## Conclusions

A simple chemical co-precipitation approach is used to make a series of single-phased Mn_*x*_Fe_1–*x*_Fe_2_O_4_ (*x* = 0–1) NPs with high crystallinity with diameters ranging from 5.78 to 9.94 nm. With Mn^2+^ substitution, the structural analysis revealed cubic spinel NPs, a higher lattice constant, and increased particle sizes. The influence of Mn^2+^ substitution on the structural and magnetic characteristics of Mn_*x*_Fe_1–*x*_Fe_2_O_4_ (*x* = 0–1) NPs is investigated, and it is found that Mn^2+^, Fe^3+^ prefers at the tetrahedral sites and Fe^2+^ the Fe^3+^ octahedral sites. In addition to saturation magnetization and remnant magnetization, the coercivity of iron oxides and manganese oxides is altered significantly by manganese incorporation due to the interaction of A- and B-site ions, which directly affect the magnetic properties. The altered magnetic properties of Mn_*x*_Fe_1–*x*_Fe_2_O_4_ (*x* = 0–1) NPs due to the distribution of cations at tetrahedral and octahedral site ultimately affects the self-heating temperature rise characteristics of MNPs. At a physiologically safe range of frequency and amplitude, the sample Mn_0.75_Fe_0.25_Fe_2_O_4_ had a maximum SAR of 153.76 W/g and ILP 1.38 nHm^2^/kg making them a suitable candidate for hyperthermia treatment.

## Supporting Information Available

Additional details of XRD, EDX, and details of the induction heating, that is, slope values calculated from the temperature *vs* time plot, and SAR values of the different concentrations of NPs at AMF of field strength (PDF)
