# Supplementary material for: Application of MnxFe1–xFe2O4 (x = 0–1) Nanoparticles in Magnetic Fluid Hyperthermia: Correlation with Cation Distribution and Magnetostructural Properties
Source: ACS Omega. 2022 Nov 22;7(48):44187–98. doi: 10.1021/acsomega.2c05651 (PMC9730757; doi:10.1021/acsomega.2c05651)
Supplement: Supplementary file 1 — ao2c05651_si_001.pdf [file ao2c05651_si_001.pdf]

Supporting information

Application of $Mn_xFe_{1-x}Fe_2O_4$ (x = 0 to 1) nanoparticles in magnetic fluid hyperthermia: Correlation with cation distribution and magneto-structural properties

Satish S. Phalake[a], Manohar S. Lad[a], Ketaki V. Kadam[a], Syed A.M.Tofail[b], Nanasaheb D. Thorat[c,b*], Vishwajeet M. Khot[a*]

[a]Department of Medical Physics, Centre for Interdisciplinary Research, D. Y. Patil Education Society (Institution Deemed to be University), Kolhapur, 416 006, MS, India
[b]Department of Physics and Bernal Institute, University of Limerick, Castletroy, Co. Limerick, Limerick, V94T9PX, Ireland
[c]Nuffield Department of Women's and Reproductive Health, John Radcliffe Hospital, Medical Sciences Division, University of Oxford, Oxford OX3 9DU, United Kingdom

Corresponding Authors
1. Nanasaheb D. Thorat[b,c*],

[b]Department of Physics and Bernal Institute, University of Limerick, Castletroy, Co. Limerick, Limerick, V94T9PX, Ireland
[c]Nuffield Department of Women's and Reproductive Health, John Radcliffe Hospital, Medical Sciences Division, University of Oxford, Oxford OX3 9DU, United Kingdom
Email: thoratnd@gmail.com; nanasheb.thorat@wrh.ox.ac.uk orcid.org/0000-0001-6343-527X

2. Vishwajeet M. Khot[a*]

Department of Medical Physics, Centre for Interdisciplinary Research,D. Y. Patil Education Society (Institution Deemed To Be University),Kolhapur, 416 006, MS, India
Email: wish.khot@gmail.com orcid.org/0000-0003-1315-9240

Table S1. Lattice constant (a), Average crystallite size by (D_{xrd}) and Transmission Electron Microscopy (D_{TEM}), X-ray density (dx), specific surface area (S) of $Mn_xFe_{1-x}Fe_2O_4$ NPs(x= 0-1)

sample (x)	a (nm)	D_{xrd} (nm)	D_{TEM} (nm)	(dx) g/cm^3	S m^2/g
**0.0**	0.8354	5.784	7.6864	5.2735	236.75
**0.25**	0.8350	5.459	8.2154	5.2762	138.44
**0.50**	0.8424	10.93	9.4810	5.1324	136.84
**0.75**	0.8436	12.92	13.603	5.1055	113.78
**1.00**	0.8409	9.945	10.213	5.1590	114.83

Energy dispersive X-ray analysis (EDX). EDX spectra for the different samples are shown in following Figure, as well as the analyzed results as shown in the respective Fig, confirming the percentage of Mn, Fe, and O elements.

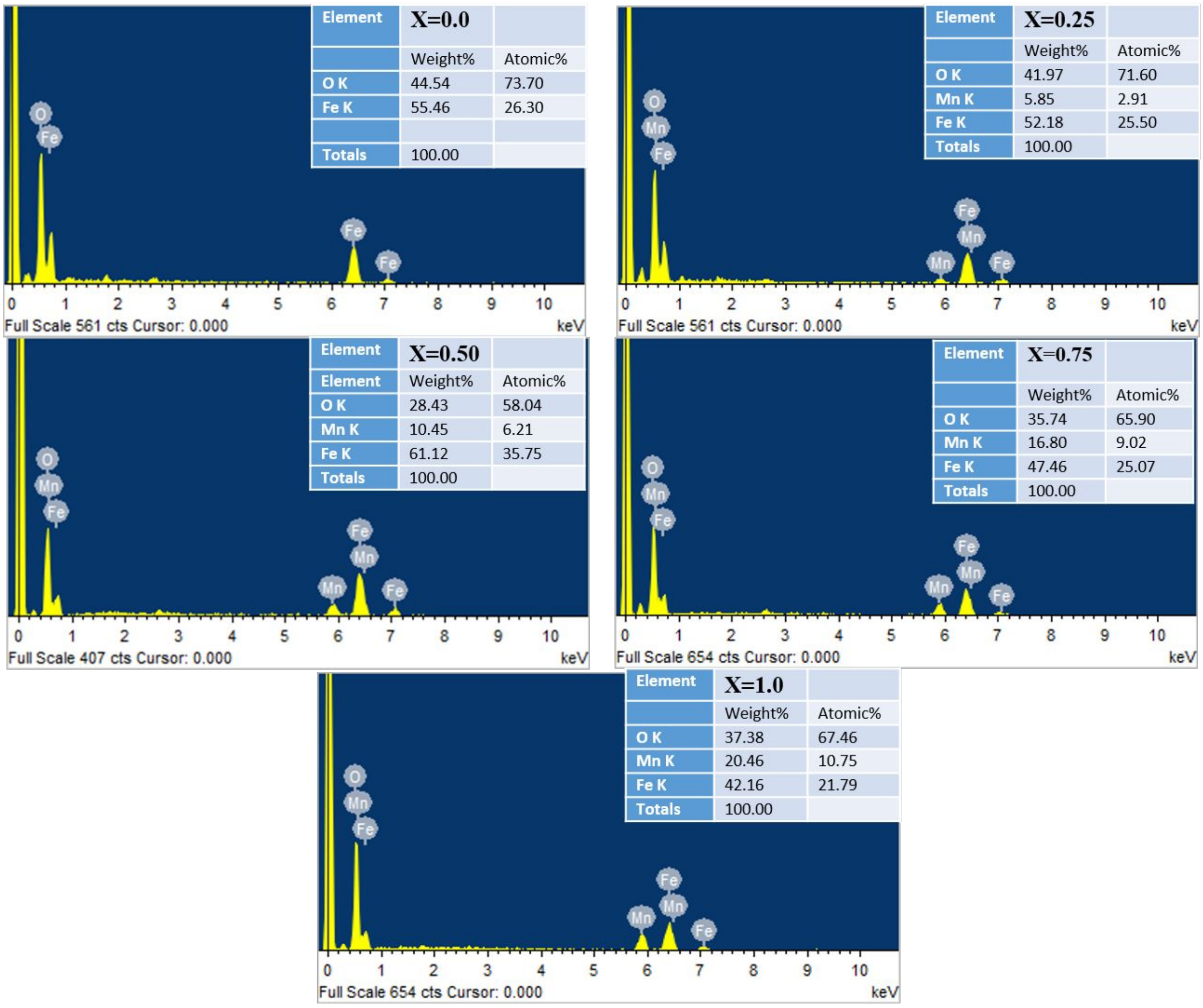

Figure S1. EDX spectra of the prepared NPs along with their respective analyzed results as given in the inset.

The temperature rises of nanoparticle with concentration (0.5, 1, 2, 5, 10 mg/ml)and field's 13.3 kA/m, 20.0 kA/m, and 26.6 kA/m.

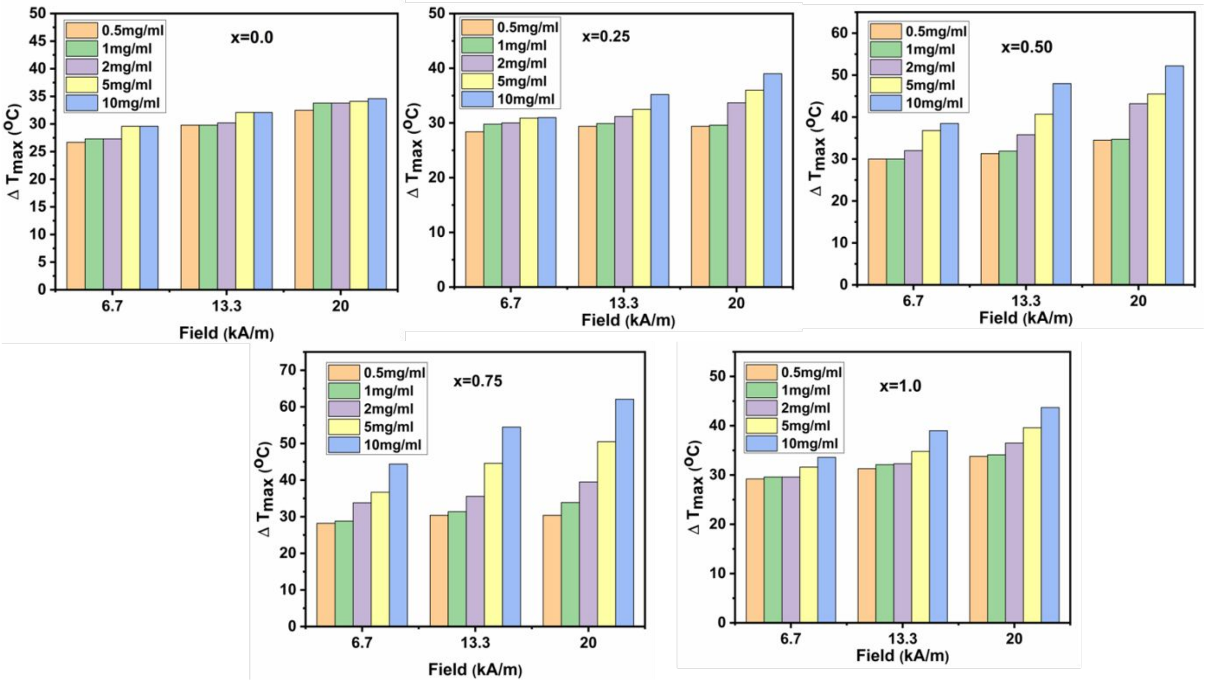

Figure S2. The growth in temperature versus time for samples at different field amplitudes.

Table S2-SAR and ILP values of $Mn_xFe_{1-x}Fe_2O_4$ (x = 0 to 1) NPs with an increase in amplitudes from 13.3 kA/m to 26.7 kA/mfor 0.5, 1, 2, 5 and 10 mg/mlrespectively.

**Applied field (kA/m) for $Mn_xFe_{1-x}Fe_2O_4$**		**specific absorption rate (SAR) W/g**					**intrinsic loss power (ILP) nHm²/kg**				
		**0.5mg/ml**	**1mg/ml**	**2mg/ml**	**5mg/ml**	**10mg/ml**	**0.5mg/ml**	**1mg/ml**	**2mg/ml**	**5mg/ml**	**10mg/ml**
***x*= 0.0**	13.3	20.95	13.96	-	-	4.9	1.12	1.68	-	-	0.39
	20.0	111.6	34.91	6.9	7.01	4.93	2.27	7.12	0.14	0.56	0.10
	26.7	139.6	76.81	17.47	12.6	10.57	1.25	0.69	0.15	0.11	0.09
***x*= 0.25**	13.3	83.76	34.91	6.99	8.41	5.63	6.7	2.80	0.56	0.67	0.45
	20.0	125.64	62.85	20.97	11.21	13.38	2.56	1.28	0.42	0.22	0.27
	26.7	107.52	125.7	24.46	29.44	16.20	1.51	1.13	0.22	0.26	0.14
***x*= 0.50**	13.3	41.88	34.91	20.97	32.25	17.61	3.36	2.80	1.68	2.59	1.41
	20.0	83.76	48.88	52.42	39.26	26.77	1.70	0.99	1.06	0.80	0.54
	26.7	125.64	97.77	76.89	47.67	29.59	1.13	0.88	0.69	0.43	0.26
***x*= 0.75**	13.3	55.84	41.90	45.43	28.04	19.73	4.49	3.36	3.65	2.25	1.58
	20.0	111.68	48.88	59.41	42.07	28.18	2.27	0.99	1.21	0.85	0.57
	26.7	153.76	76.81	87.12	43.47	33.11	1.38	0.69	0.78	0.88	0.67
***x*= 1.0**	13.3	41.88	20.95	17.47	11.21	7.75	3.36	1.68	1.40	0.90	0.62
	20.0	69.80	34.91	20.97	16.82	16.20	1.42	0.71	0.42	0.34	0.33
	26.7	125.64	76.81	41.94	43.47	32.41	1.13	0.69	0.37	0.39	0.29
